# Multimodal Fusion-Based Self-Calibration Method for Elevator Weighing Towards Intelligent Premature Warning

**DOI:** 10.3390/s25175550

**Published:** 2025-09-05

**Authors:** Jiayu Luo, Xubin Yang, Qingyou Dai, Weikun Qiu, Siyu Nie, Junjun Wu, Min Zeng

**Affiliations:** 1 South China Academy of Advanced Optoelectronics, South China Normal University, No. 378, Waihuan West Road, Panyu District, Guangzhou 510006, China; 2023024189@m.scnu.edu.cn (J.L.); zengmin@scnu.edu.cn (M.Z.); 2 Guangdong Institute of Special Equipment Inspection and Research Foshan Branch, No.2, Yingyin 2 Street, Chancheng District, Foshan 528012, China; 13702561188@139.com; 3 Key Laboratory of the State Administration for Market Regulation, Guangdong Institute of Special Equipment Inspection and Research, No. 111, Huandao South Road, Nanhai District, Foshan 528251, China; daiqingyou@126.com; 4 Guangdong Provincial Key Laboratory of Industrial Intelligent Inspection Technology, School of Mechatronic Engineering and Automation, Foshan University, No.33 Guangyun Road, Shishan Town, Nanhai District, Foshan 528200, China; 2112351002@fosu.stu.edu.cn (W.Q.); 2112451130@fosu.stu.edu.cn (S.N.)

**Keywords:** elevator load weighing, self-calibration, multimodal information fusion

## Abstract

As a high-frequency and essential type of special electromechanical equipment, a vertical elevator has a significant societal implication for their safe operation. The load-weighing module, serving as the core component for overload warning, is susceptible to precision degradation due to the nonlinear deformation of rubber buffers installed at the base of the elevator car. This deformation arises from the coupled effects of environmental factors such as temperature, humidity, and material aging, leading to potential safety risks including missed overload alarms and false empty status detections. To address the issue of accuracy deterioration in elevator load-weighing systems, this study proposes an online self-calibration method based on multimodal information fusion. A reference detection model is first constructed to map the relationship between applied load and the corresponding relative compression of the rubber buffers. Subsequently, displacement data from a draw-wire sensor are integrated with target detection model outputs, enabling real-time extraction of dynamic rubber buffers’ deformation characteristics under empty conditions. Based on the above, a displacement-based compensation term is derived to enhance the accuracy of load estimation. This is further supported by a dynamic error compensation mechanism and an online computation framework, allowing the system to self-calibrate without manual intervention. The proposed approach eliminates the dependency on manual tuning inherent in traditional methods and forms a highly robust solution for load monitoring. Field experiments demonstrate the effectiveness of the proposed method and the stability of the prototype system. The results confirm that the synergistic integration of multimodal perception and adaptive calibration technologies effectively resolves the challenge of load-weighing precision degradation under complex operating conditions, offering a novel technical paradigm for elevator safety monitoring.

## 1. Introduction

Vertical elevators are indispensable vertical transportation devices in modern urban buildings, and their stability, safety, and maintenance quality constitute an essential part of the urban public safety system. Particularly with the rapid development of high-rise and super high-rise buildings, the frequency of elevator operation has increased significantly, imposing higher requirements for real-time and accurate load state perception [1]. However, existing elevator load monitoring methods are predominantly based on mechanical measurement or single-sensor schemes, which suffer from limitations such as insufficient measurement accuracy, poor environmental adaptability, and high maintenance costs. In particular, after prolonged use, the aging of rubber buffers and the influence of complex external environmental factors lead to a marked decline in the stability and accuracy of traditional weighing systems, making it difficult to effectively address the safety risks caused by missed overload alarms and false empty status detections. Therefore, there is an urgent need to explore more reliable and intelligent load monitoring technologies. To address the above challenges, this study proposes a multimodal elevator weighing method that integrates displacement sensing with visual perception. By employing an embedded intelligent terminal, the system enables the real-time and accurate analysis of the load state, achieving dynamic self-calibration and overload identification. This approach effectively improves the measurement accuracy and operational stability and demonstrates strong application potential in enhancing elevator safety management, energy efficiency, and maintenance performance.

## 2. Related Works

Elevator load monitoring technology has long relied on single-sensor solutions such as pressure sensors and strain gauges. However, in complex operational environments and under long-term service conditions, these systems face significant challenges, including degraded monitoring accuracy and insufficient real-time performance [2,3,4]. Recent research efforts have primarily focused on three directions: multi-sensor data fusion, intelligent algorithm optimization, and remote monitoring.

In China, several advancements have been made. Hangzhou Ambida Elevator Co., Ltd. developed a load-weighing device based on the mechanical deformation detection of steel wire ropes, which indirectly measures the load through a spring structure and provides overload warnings. Nevertheless, this approach is susceptible to spring fatigue failure and delayed dynamic response [2]. Shanghai Mitsubishi Elevator Co., Ltd. employs absolute position sensors to estimate the car load by measuring the vertical displacement, simplifying the installation but requiring precise calibration of multiple elastic coefficients [3]. Shandong Fuji Control Electric Co., Ltd. integrates pressure and displacement sensors to monitor the rope-head load. However, its averaging mechanism across multiple sensors may compromise the system’s robustness [4]. Toshiba Elevator (China) Co., Ltd. introduced a CAN bus-based hierarchical load monitoring scheme that improves communication efficiency but suffers from increased integration complexity, which limits the dynamic response speed [5]. Schindler China Elevator Co., Ltd. utilizes a central pressure sensor to directly measure the load in belt-type elevators, though the use of a single sensing unit makes the system vulnerable to load eccentricity and reduced stability [6]. Haomen Electronic Technology (Xiamen) Co., Ltd. proposed a novel integration of dynamic weighing units with vision-based detection, but the structural complexity increases the maintenance cost and environmental sensitivity [7].

In other countries, research has focused on enhancing the accuracy and system intelligence. The Otis Elevator Company deployed a distributed pressure sensor array to achieve high-precision load measurements, but the multi-sensor layout increases the system complexity [8]. KONE established a sensor network to support predictive maintenance, though its reliance on algorithms and vulnerability to environmental interference limit real-time performance [9]. Thyssenkrupp Elevator developed a compact indirect measurement system based on rope tension sensing, which is sensitive to rope wear conditions [10]. Mitsubishi Electric integrated pressure sensors with optical detection to improve the load distribution identification; however, the limited environmental adaptability of optical units remains a major implementation bottleneck [11]. Hitachi introduced accelerometers to enhance the dynamic load response, though vibration interference poses a significant source of measurement error [12].

In recent years, multi-sensor fusion technology has demonstrated significant potential in elevator monitoring, opening up new research directions for intelligent solutions. Guo et al. [13] proposed a real-time elevator fault monitoring system that combines vibration and displacement data, enabling cloud-based data access. However, the self-calibration and dynamic error compensation issues of load weighing have not yet been addressed. Kullu and Cinar [14] developed a deep learning method that fuses vibration and current data, significantly improving the accuracy of industrial equipment fault detection. However, this method is not optimized for elevator load monitoring scenarios. These studies have demonstrated the effectiveness of multimodal fusion under complex operating conditions but remain limited in addressing accuracy drift under dynamic loads. Khatir et al. [15] systematically reviewed the applications of machine learning and deep learning in structural health monitoring, highlighting their advantages in processing complex data and achieving real-time monitoring, providing theoretical support for the design of multimodal fusion monitoring systems. Garcia-Perez et al. [16] explored the performance of edge computing devices in embedded AI tasks, demonstrating their efficiency in resource-constrained environments. This is highly consistent with the design goal of this study, which aims to achieve low-power real-time monitoring based on the Rockchip RK3568 platform. Dilmi et al. [17] compared the performance of YOLOv5 and YOLOv8 on embedded platforms. The results showed that YOLOv8 demonstrated a better balance between accuracy and real-time performance in embedded environments, providing strong support for the selection of visual detection models in this study.

Overall, the current research suffers from three critical limitations: (1) single-sensor schemes are unable to effectively resist the accuracy drift caused by environmental disturbances and mechanical aging; (2) multi-sensor systems often sacrifice reliability due to their structural complexity; (3) real-time error compensation mechanisms under dynamic load scenarios remain underdeveloped. These gaps provide opportunities for innovations that integrate multi-source sensing with online self-calibration techniques. It is noteworthy that the use of car-base buffer rubber is a common practice in the elevator industry. However, for the vast existing and incremental equipment, no effective self-calibration method has been reported. The self-calibration approach proposed in this paper is an industry first, demonstrating significant advancements, with its effectiveness fully validated in Section 3.

## 3. System Architecture Design

**Figure 1:**
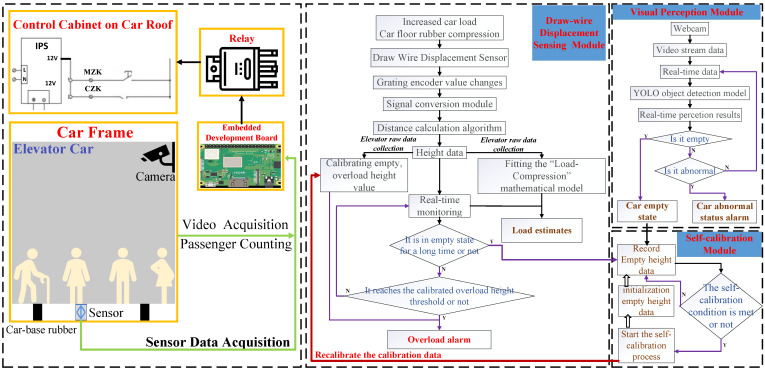
Overall system architecture and technical roadmap.

As shown in Figure 1, the system establishes a dynamic elevator load monitoring framework based on multi-sensor fusion and adaptive control technologies. Its core functions are realized through the collaboration of the draw-wire displacement sensing module, visual perception module, and intelligent self-calibration module. Each module contributes to accurate load perception and error compensation through a closed-loop data flow. The core functionalities of each module are illustrated in Figure 1:

### 3.1. System Architecture and Core Module Design

1. **The draw-wire displacement sensing module** is primarily responsible for monitoring the deformation of the rubber buffers located at the base of the elevator car (hereinafter referred to as “car-base rubber”). Based on the analysis of the elevator structural parameters, operational characteristics, and mechanical force transmission mechanisms, the module captures the displacement variation patterns induced by loading and constructs a mapping model between the applied load and relative compression, enabling online estimation of the elevator car load.
2. **The visual perception module** adopts a lightweight object detection model, deployed on an edge computing platform, to achieve high-accuracy and low-latency tasks such as passenger counting, empty state identification, and abnormal behavior detection under constraints of spatial and computational resources. It provides prior information from the visual modality for the self-calibration module.
3. **The intelligent self-calibration module** performs integrated analysis of data from both the displacement sensor and the visual module to conduct dynamic error correction and anomaly identification. When the elevator car is in a empty and stationary state, this module records the current height value from the displacement sensor and analyzes in real time whether the reference point has drifted. Based on this analysis, it determines whether aging or deformation has occurred in the car-base rubber. The system then adaptively adjusts the overload warning threshold to compensate for the sensor measurement deviation caused by rubber material degradation, thereby improving the long-term operational stability of the system under high-frequency usage conditions.

### 3.2. Core Module Design

#### 3.2.1. Load Estimation Method

To address the lack of real-time load information in conventional elevators, a load estimation method is designed that combines a physical mapping between the load and relative compression with piecewise linear interpolation, based on a draw-wire displacement sensor. This method utilizes the structural configuration of the draw-wire displacement sensor to measure the deformation of the car-base rubber elastomer. By integrating the measurement results with the calibration samples, a set of physical-to-data mapping nodes is constructed, enabling the formulation of a load–relative compression mapping model for the real-time estimation of the elevator car load.

When subjected to loading, the car-base rubber undergoes deformation, and the draw-wire displacement sensor is capable of continuously monitoring this compression. According to the equivalent elastic response described by Hooke’s Law, a relationship exists between the compression and the applied force (load) within the working range of the car-base rubber, expressed as follows:

(1) F≈k·ΔH.

Among them, *F* is the car load, ΔH is the car-base rubber compression, and *k* is the equivalent stiffness. The actual car-base rubber material shows a certain nonlinearity, and the parameter *k* varies in different elevator systems and different aging states. Therefore, using measured samples to calibrate the “load–relative compression” is the basis for ensuring the accuracy of the estimation.

In the load estimation model construction phase, it is first necessary to record the absolute height value returned by the draw-wire displacement sensor when the car is empty as the reference value $H_{0}$, and then obtain the corresponding compression $\Delta H_{i}$ under different known loads $F_{i}$ by loading a series of standard weights. The measured point set is organized into a load–relative compression mapping calibration node set:

(2) $$\{\left(\Delta H_{1},F_{1}\right),\left(\Delta H_{2},F_{2}\right),\ldots ,\left(\Delta H_{n},F_{n}\right)\}.$$

During the load estimation process, the relative compression can be calculated by using the real-time height data $H_{abs}$ and the reference value $H_{0}$ obtained by the draw-wire displacement sensor at the base of the car:

(3) $$\Delta H=H_{0}-H_{abs}.$$

Considering the actual nonlinearity and the limited resources of the embedded controller, a piecewise linear interpolation model is used to establish the global load estimation model. For any compression input ΔH, first locate the interval $\left[\Delta H_{i},\Delta H_{i+1}\right]$ in which it falls, and then use the linear interpolation formula to calculate the estimated load:

(4) $$F\left(\Delta H\right)=F_{i}+\frac{\Delta H-\Delta H_{i}}{\Delta H_{i+1}-\Delta H_{i}}\left(F_{i+1}-F_{i}\right).$$

This formula ensures the accurate fitting of the original observation data at the calibration node and achieves smooth estimation within the interval. The entire estimation process only requires basic addition, subtraction, multiplication, and division operations, which is very suitable for efficient implementation in embedded systems. The first-order derivative of each segment of the model is

(5) $$k_{i}=\frac{F_{i+1}-F_{i}}{\Delta H_{i+1}-\Delta H_{i}},\;\left(i=0,\ldots ,49\right).$$

$k_{i}$ represents the equivalent stiffness of the material in this range. The model can be dynamically adjusted with factors such as material aging, temperature, and humidity environment, which is convenient for subsequent self-calibration or parameter update.

In summary, the theoretical core of this method is to take physical modeling as the basis, combine piecewise linear interpolation with material nonlinearity and individual differences of the system, and ensure estimation accuracy while achieving computational efficiency and model scalability. It can provide low-cost highly reliable real-time load monitoring capabilities for various vertical elevator systems without increasing additional hardware costs.

#### 3.2.2. Visual Module Design

To enhance the system’s perception of the elevator car occupancy status and assist in triggering the empty judgment and self-calibration logic, a visual intelligence module is designed in this study, achieving millisecond-level response after deployment on the embedded platform. Since the primary focus of this work is to develop a reliable online self-calibration mechanism for overload warning, aimed at addressing the problem of inaccurate elevator load monitoring, the target detection algorithm itself is not the main subject of investigation. Inspired by the related work [18,19], considering the detection accuracy, real-time performance, and compatibility with edge computing platforms, YOLOv8 [20] is adopted in this study to infer information such as the empty status and passenger count inside the elevator car, serving as an auxiliary input to the self-calibration mechanism.

The YOLOv8 model uses the Anchor-Free mechanism, and the output result is a target set:

(6) $$y=\{c_{i},b_{i},s_{i}|i=1,2,\ldots ,N\},$$

where $c_{i}\in Z^{+}$ is the i-th target category (such as “passenger”), $b_{i}=\left(x_{i},y_{i},w_{i},h_{i}\right)$ represents the center position and width and height of the bounding box, and $s_{i}\in \left[0,1\right]$ is the confidence score. The final number detection result is recorded as

(7) $$N=\sum_{i=1}^{N}I\left(c_{i}=\mathrm{passenger}\wedge s_{i}\geq 0\right),$$

where I(·) is the indicator function, and θ is the confidence threshold (taken as 0.85).

During the model training process, Mosaic data enhancement and CIoU Loss are introduced to improve the target box regression accuracy, which is defined as follows:

(8) $$L_{cloU}=1-IoU+\frac{\rho^{2}\left(b,b^{*}\right)}{c^{2}}+\alpha v,$$

where ρ(·) represents the Euclidean distance between the center point of the predicted box and the true box, *c* is the length of the diagonal, and α and *v* are used to measure the consistency of the aspect ratio, which helps to improve the convergence speed and accuracy stability.

After model training, the network is exported in ONNX format and quantized using INT8 post-training quantization (PTQ) via the RKNN toolchain. The quantized model is then deployed to the NPU unit of the embedded development board to perform inference tasks [21]. The system adopts a collaborative ARM + NPU architecture, in which the ARM core handles image preprocessing and postprocessing, while the NPU executes the forward computation of the backbone neural network, thereby ensuring real-time detection performance [22].

The number of people *N* output by this module will be used together with the sensor estimation to determine whether the elevator is in an empty and stationary state, satisfying:

(9) $${\mathrm{Trigger}}_{\mathrm{calib}}=I\left(N=0\wedge T_{\mathrm{stable}}\geq T_{0}\right).$$

Among them, $T_{stable}$ represents the length of time that the displacement data remain stable, and $T_{0}$ is the minimum trigger time (taken as 20 min).

In summary, this module is guided by engineering deployment and combines data collection, model training, quantitative deployment, and edge reasoning to build a complete workflow, which not only improves the comprehensiveness and accuracy of the cabin status perception but also provides key auxiliary support for subsequent self-calibration logic and abnormal alarm.

#### 3.2.3. Intelligent Self-Calibration Method

Conventional elevator overload detection systems typically rely on mechanical micro-switches and fixed overload thresholds to determine the full-load condition. However, with long-term operation, aging and degradation of the compression performance of the car-base rubber lead to systematic drift in deformation response, which in turn causes false positives or missed detections in overload alarms. To improve the long-term stability and maintainable accuracy of the system, the proposed method replaces the original micro-switch with a draw-wire sensor to perform distance monitoring. An embedded system continuously monitors sensor data during empty and stationary conditions and applies a multi-cycle averaging comparison algorithm to automatically identify the aging status of the car-base rubber. The full-load threshold is dynamically updated and compensated accordingly. This method not only retains the original overload protection functionality but also introduces load estimation and automatic compensation for overload alarm offset due to mechanical degradation.

During normal operation of the elevator, when Equation (9) is met, the system considers that the elevator is in an empty state. At this time, the calculation control module starts to record multiple draw-wire sensor height samples $\left\{H_{1},H_{2},\ldots ,H_{n}\right\}$ in the empty state and calculates the zero-load height average $H_{a}$ once a week. If the difference in the equivalent load drift corresponding to $\left|H_{a}-H_{0}\right|$ exceeds the set allowable deviation, the calculation control module determines that the car-base rubber is aging, and the car full load height needs to be recalibrated. The latest zero load height average is used to update the old zero load height, and the difference between the two is compensated for the full load height to form a new zero load height $H_{0}^{\prime }$ and full load height $H_{m}^{\prime }$, that is

(10) $$H_{0}^{\prime }=H_{a}=\frac{\sum_{i=1}^{n}H_{i}}{n}$$

(11) $$H_{m}^{\prime }=H_{m}-\left(H_{0}-H_{a}\right).$$

**Figure 2:**
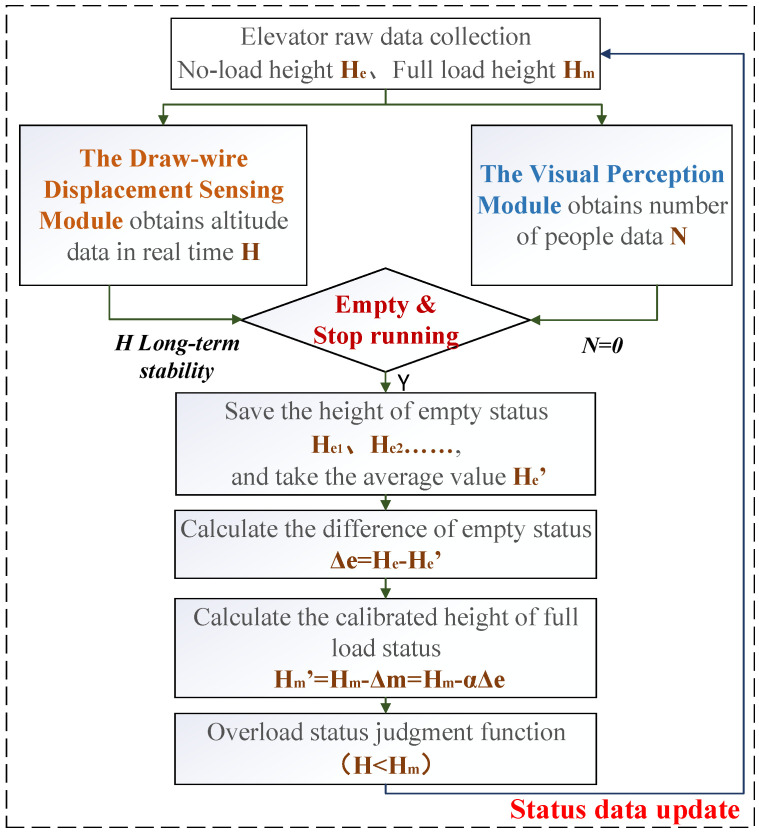
Flowchart of the self-calibration procedure.

As shown in Figure 2, the self-calibration procedure flow updates the data by clearing the previously recorded Hi and applies the newly obtained empty height and full-load height for subsequent full-load condition judgments in daily elevator operation. This method enables the system to achieve a closed-loop process of self-perception, self-correction, and self-adaptation in response to material aging and internal error drift. It eliminates the high cost and operational downtime risks associated with the manual load calibration in traditional approaches, significantly enhancing the engineering practicality and lifecycle intelligence level of the system.

## 4. Experimental Evaluation and Application Validation

### 4.1. Hardware Deployment and Testing Platform

**Figure 3:**
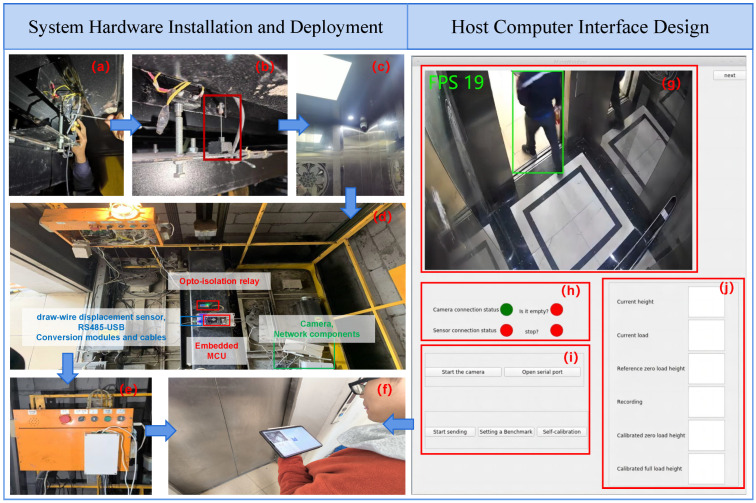
Application validation test platform: hardware deployment and human–machine interface. (**a**,**b**) show the draw-wire displacement sensor. (**c**) shows the camera of the visual perception module. (**d**,**e**) shows the core hardware and modules in an enclosure. (**g**) shows the real-time monitoring. (**h**,**j**) show the system status feedback. (**i**) shows the control buttons. (**f**) shows a scenario of remote monitoring and debugging.

As illustrated in Figure 3, this section presents the application test deployment of the elevator car weighing system and its integrated human–machine interface.

In Figure 3a,b, the draw-wire displacement sensor is vertically mounted at the central rigid support point of the car base with bolts. The sensor’s axis is adjusted to align vertically with the car’s movement trajectory, thereby ensuring the accuracy of the displacement data acquisition. Figure 3c shows the vision perception module fixed to the car ceiling at a 30° downward angle. It connects to a PoE switch via a shielded CAT6 cable, establishing a Local Area Network (LAN) with the embedded development board. The same-subnet design minimizes the communication latency, enabling reliable real-time video transmission. The embedded development board acquires data from the draw-wire displacement sensor. An opto-isolated relay implements control functions with galvanic isolation between low-voltage and high-voltage circuits. As shown in Figure 3d,e, the core modules are integrated within a structural enclosure, preventing direct contact with the high-voltage components and enhancing the operational safety.

Figure 3g–j demonstrate the intelligent module developed on the embedded Qt platform, which supports remote access and Over-The-Air (OTA) updates. The graphical interface integrates functionalities for real-time monitoring, system status feedback, data interaction, and historical records, dynamically displaying key parameters including the passenger count, car load, and calibration status. Maintenance personnel can perform cross-platform remote monitoring and program upgrades via multiple terminals (Figure 3f), significantly improving the quality, efficiency, and safety of intelligent operation and maintenance.

### 4.2. Load Estimation Model Initialization

First, the absolute height value returned by the draw-wire sensor when the car is empty is recorded as the baseline value ($H_{0}$) for that particular round of data collection. Subsequently, standard 20 kg weights are incrementally added to the empty car until reaching the full load of 1000 kg. After each load step stabilizes for 3 s, the absolute height value ($H_{abs}$) is recorded. This process collects 51 data points sequentially. Following this, all weights are removed, the baseline is reset, and this procedure is repeated to collect 5 rounds of raw data, resulting in a total of 255 raw data pairs. For each round of raw data, the relative compression (ΔH) corresponding to each load level is calculated relative to that round’s baseline value $H_{0}$ using Equation (3). Subsequently, the arithmetic mean of the ΔH for the same load level across different rounds is computed to reduce the noise, forming a set of 51 calibration nodes:

(12) $$\left(\Delta H_{i},F_{i}\right),F_{i}=20i,\;i=0,1,2,\ldots ,50,$$

where $\Delta H_{i}$ represents the corresponding compressive displacement (mm), which strictly monotonically increases from 0 mm to 3.08 mm; $F_{i}$ denotes the load magnitude (kg).

**Figure 4:**
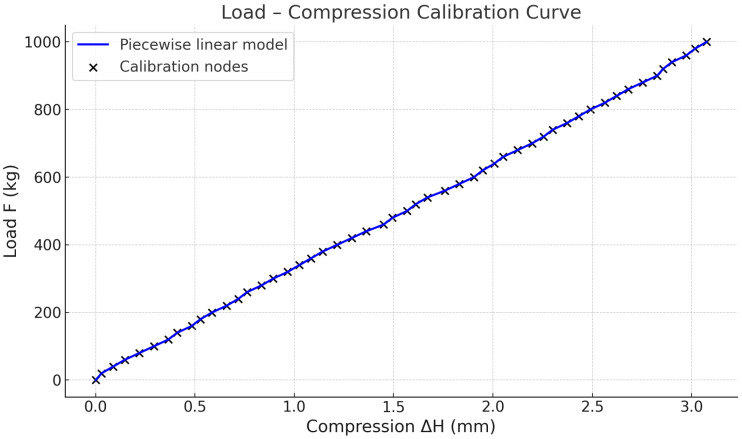
Calibration node fitting curve.

As indicated by these calibration nodes, the load exhibits an approximately linear relationship with the relative compression (as shown in Figure 4). Considering the limited computational resources of the embedded controller, this study employs piecewise linear interpolation to establish the load estimation function. For any measured $\Delta H\in (\Delta H_{i},\Delta H_{i+1})$, the corresponding load *F* can be estimated using Equation (4).

### 4.3. Dataset Construction

**Figure 5:**
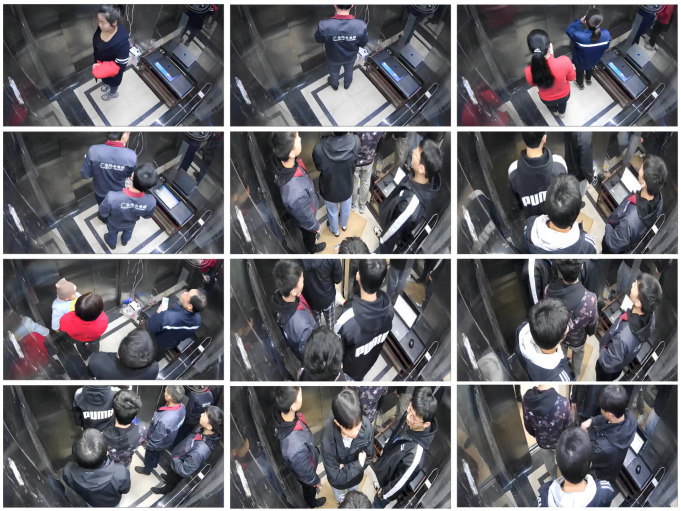
Examples of elevator image dataset.

To address the challenge of passenger detection in complex elevator car environments, characterized by illumination variations, viewpoint changes, dense target occlusion, and overlap, this study employed a fixed ceiling-mounted camera (1920 × 1080 resolution @ 30 fps). Diverse images were captured under varied lighting conditions and with different passenger groups, thereby constructing an evaluation dataset comprising 4568 images. As illustrated in Figure 5, this dataset mitigates the critical issue of data scarcity for samples inside vertical elevator cars, representing a significant contribution to the research domain of elevator intelligence.

The operational condition distribution of the dataset was meticulously designed to reflect the diversity of real-world elevator scenarios: 50% of the images (2284) capture multi-passenger load scenarios, 40% (1827) correspond to empty cabin states, and the remaining 10% (457) encompass complex conditions, including dense occlusion, rapid passenger entry/exit, dynamic scenes during door opening/closing, and interference from specular reflections and adverse lighting. This distribution ensures robust model performance and generalization across diverse operational contexts.

To ensure annotation accuracy and consistency, all images were manually annotated using the LabelMe tool, with cross validation by multiple annotators to minimize errors. The dataset was subsequently partitioned into a training set (3654 images) and a validation set (914 images) in an 8:2 ratio and converted into the COCO format, facilitating the training and performance evaluation of the YOLOv8 object detection model.

To tackle noise and data quality challenges in complex environments, systematic preprocessing and augmentation were applied to the raw images prior to training. The preprocessing steps included resizing images to 640 × 640 pixels to align with YOLOv8’s default input size, converting the color space from BGR to RGB, and normalizing the pixel values to the [0,1] range by dividing by 255 to meet standardized model input requirements. Data augmentation adopted YOLOv8’s default strategies, including random horizontal flipping, brightness and saturation jittering in HSV color space, and Mosaic multi-image stitching to simulate dense target scenarios, thereby enhancing the model robustness against viewpoint changes and occlusions while mitigating overfitting risks. Furthermore, YOLOv8’s training pipeline automatically incorporates affine transformations (e.g., random scaling, translation, and rotation) and additional color perturbations, bolstering model generalization without requiring further configuration.

### 4.4. Effectiveness Evaluation of the Visual Module

To evaluate the accuracy of the visual module in identifying the empty state and counting passengers within the elevator car during actual operation, this study designed experiments specifically for empty status detection and passenger counting. The detailed test procedure is as follows: During routine elevator operation, video data of the car interior were randomly captured, resulting in a total of 1500 representative experimental sample frames. Among these samples, 804 frames depict the empty state, while the remainder represent loaded states with the number of passengers ranging from one to five persons. All collected samples were subsequently input into the visual module deployed on the LubanCat 2 edge computing platform, which is equipped with a Rockchip RK3568 processor featuring a quad-core ARM Cortex-A55 CPU, an integrated 0.8 TOPS NPU, 4 GB LPDDR4 memory, and support for USB 3.0, GPIO sensor expansion, and Mini-PCIe interfaces. The system automatically outputs, for each frame, the empty status detection result and the passenger count value. Using manual annotation as the reference standard, the system’s determinations were compared against the ground truth. The number of correct identifications was then separately tallied for the empty state and for passenger counts. The evaluation metrics are described as follows:

- Empty State Recognition Accuracy
  (13) $$AC_{\mathrm{empty}}=\frac{N_{\mathrm{correct}}}{N_{e}}\times 100\%,$$
  where $N_{correct}$ is the number of frames in which the empty state was correctly identified, and $N_{e}$ is the total number of empty sample frames.
- Passenger Counting Accuracy
  (14) $$AC_{\mathrm{count}}=\frac{N_{\mathrm{cc}}}{N}\times 100\%,$$
  where $N_{cc}$ is the number of frames with correctly counted passengers, and *N* is the total number of sample frames.

Based on experimental validation under diverse real-world conditions, the visual module achieves a high recognition accuracy of 98.9% in detecting the empty state of the elevator car (as illustrated in Table 1). This result indicates that the probability of misclassifying an empty cabin is extremely low in practical applications, thereby ensuring the reliability of the self-calibration trigger condition. For the passenger counting task inside the elevator cabin, the visual module attains an accuracy of 93.5%. Although this is slightly lower than the empty-state recognition accuracy, it remains at a high level considering the complex and dynamic environments. This reflects the robustness and engineering viability of the module. Benchmark tests show that the vision model exhibits strong real-time performance on the LubanCat 2 platform. For a 640 × 640 resolution input image, the average inference time is 45 ms per frame, with end-to-end processing (including preprocessing and postprocessing) achieving 22 FPS. Under varying load conditions, the inference latency ranges from 30–60 ms, with power consumption remaining stable at 6–8 W during extended operation. Compared to the CPU-only mode (latency exceeding 200 ms), the INT8 quantized model on the NPU achieves approximately 4–5 times acceleration. This performance validates the model’s efficiency on embedded hardware and ensures its seamless integration into the proposed multimodal fusion and self-calibration framework, meeting the real-time requirements for elevator safety monitoring.

**Figure 6:**
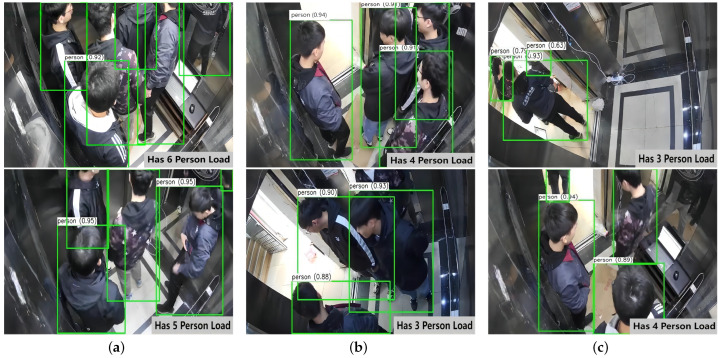
Analysis of misidentification under multiple Factors. (**a**) Mirror reflections, (**b**) occlusion, and (**c**) interference from outside passengers.

In addition, approximately 5.2% of the total samples exhibited misclassification. Further analysis reveals that these misidentifications are caused by three categories of challenging scenarios (as illustrated in Figure 6):

- Ghosting effects caused by reflections in the car’s mirrors.
- Target missed detection due to severe occlusion by overcrowded passengers.
- Extraneous pedestrians outside the elevator door being captured into the frame during door openings.

These specific misclassification scenarios account for a substantial proportion of the total errors and represent the primary factors limiting further improvements in the recognition accuracy. It is worth noting that the self-calibration function is only activated when the elevator is stationary, empty, and the doors are closed. Therefore, the misclassification types shown in Figure 6 do not have any substantive impact on the core functionality of the proposed system.

### 4.5. Effectiveness Evaluation of the Load Estimation Module

To assess the load estimation accuracy and stability of the system under varying operating conditions, this study designed simulation experiments for two typical scenarios. The process of passengers entering the elevator car was simulated by quantitatively adding and removing weights, thereby creating environments for “Stable empty loading experiments” and “Unstable empty loading experiments” to evaluate the system’s weight detection error. The specific descriptions are as follows.

#### 4.5.1. Stable Empty Loading Experiment

This experiment simulates the typical condition where passengers enter after the elevator has remained empty and stationary for an extended period. It tests the system’s load estimation accuracy under the condition that the car-base rubber has fully rebounded, and the deformation response is stable. The specific test procedure is as follows: Confirm the elevator is in an empty and stationary state, and allow the system to remain idle for more than 10 min. Record the current encoder value of the sensor as the baseline value for empty state ($H_{0}$) for the current test group. Load standard weights equivalent to $F_{true}=50$ kg into the car within 5 s. Wait until the system’s estimated output value stabilizes, then record the estimated load ($F_{est}$). Unload the weights and wait for the sensor data to return to $H_{0}$. Sequentially increase the $F_{true}$ to 50 kg, 100 kg, 150 kg, …, 1000 kg. For each load level, record both the true load value ($F_{true}$) and the model’s estimated value ($F_{est}$). Repeat the above procedure to obtain eight sets of data.

This scenario can be used to verify the accuracy baseline of the model under relatively stable operating conditions. The piecewise linear interpolation model was employed for load estimation. The Mean Absolute Error (Mean AE) and Maximum Absolute Error (Max AE) metrics were used to evaluate the experimental results:

(15) $$AE=\;|F_{est}-F_{\mathrm{true}}|,\;\mathrm{Mean}\;AE=\frac{1}{n}\sum_{j=1}^{n}AE_{j},\;\mathrm{Max}\;AE=\max\left(AE_{j}\right).$$

**Figure 7:**
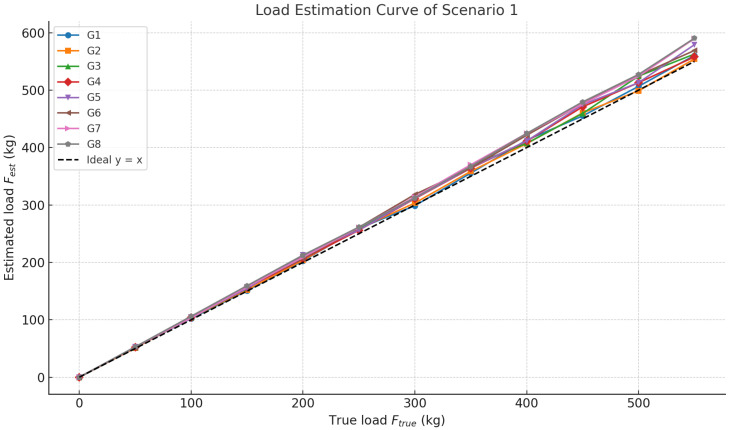
Load estimation curve of Scenario 1.

As shown in Figure 7, the experimental data indicate that in the low-load range (0–400 kg), the estimated values from all test groups closely match the actual load values, demonstrating that the deformation behavior of the car-base rubber within this range is approximately linear and that the load estimation model achieves a high degree of fitting accuracy. In contrast, in the high-load range (600–1000 kg), deviation in the estimated values is observed, which is primarily caused by the nonlinear physical deformation characteristics of the rubber material, as well as by compounded environmental disturbances.

As summarized in Table 2, the Mean AE across all test groups falls within the range of 9.11 to 16.9 kg, while the Max AE ranges from 30.3 to 47.45 kg, predominantly occurring in the high-load interval of 800–1000 kg. It is worth emphasizing that even under conditions where estimation errors are more pronounced, the maximum observed error remains below the typical single-passenger weight standard defined in the elevator industry. This result confirms the strong engineering applicability and safety redundancy of the proposed load estimation model.

#### 4.5.2. Unstable Empty Loading Experiment

This experiment simulates scenarios where passengers re-enter the elevator immediately after short unloading periods. Under such conditions, the car-base rubber lacks sufficient rebound time, causing hysteresis in the displacement sensor response that may introduce estimation deviations. The specific test procedure is as follows: Confirm the elevator is in an empty stationary state, and record the draw-wire sensor reading as the initial height ($H_{0}$). Load masses $F_{pre}\in \left\{100,200,\ldots ,1000\right\}$ kg into the car, and maintain the preload for a dwell time $T_{pre}\in \left\{0.5,1.0,1.5,2.0\right\}$ min. Unload $F_{pre}$ completely; then, reload $F_{true}\in \left\{200,400,600,800\right\}$ kg within 5 s, and record the system’s estimated output. Repeat the above procedure to obtain 40 sets of data.

This scenario evaluates the impact of elastic hysteresis on the estimation accuracy. The evaluation uses the AE and Max AE metrics defined in Equation (15).

**Figure 8:**
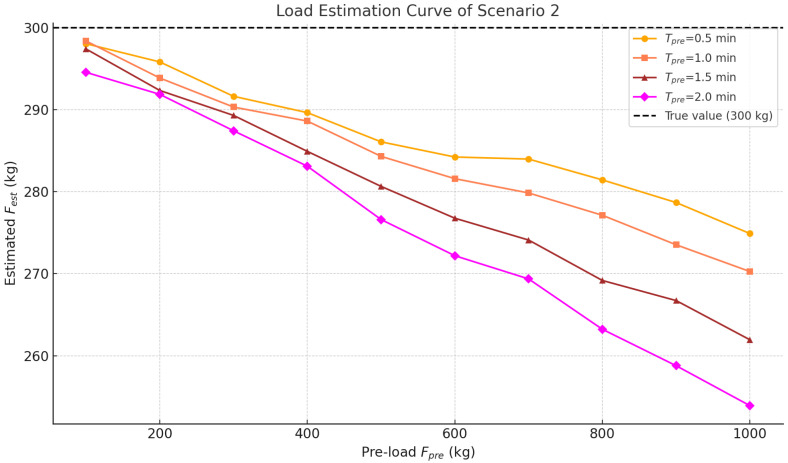
Load estimation curve of Scenario 2.

**Figure 9:**
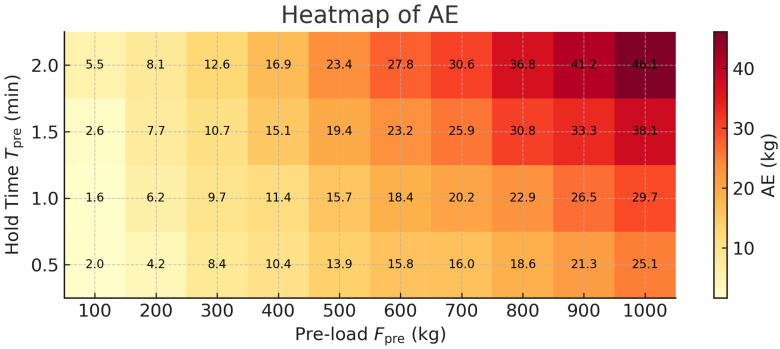
Estimation error heatmap of Scenario 2.

As shown in Figure 8, the experimental data reveal that the larger the preload mass and the longer the preload duration, the more pronounced the deviations in the estimated values across different test groups. The AE for each group is presented in Figure 9, where the maximum error reaches 46.1 kg under the extreme condition ($F_{pre}=1000$ kg, $T_{pre}=2.0$ min).

The results indicate that when the preload mass is small and the preload duration is short, the load estimation exhibits higher accuracy and remains consistently stable. Under more extreme conditions—specifically large preload masses combined with extended durations—the estimation error increases slightly. This phenomenon reflects the elastic hysteresis effect of rubber materials, wherein the deformation is not fully recovered immediately after unloading, leading to a shift in sensor readings and a subsequent reduction in estimation accuracy during reloading. Nevertheless, the overall magnitude of the estimation errors remains within acceptable industry limits, consistently falling below the standard body weight of a single adult. In summary, this section confirms both the effectiveness and robustness of the load estimation module under dynamic and complex loading fluctuations. Moreover, when applied to overload warning scenarios, the residual estimation error can be further mitigated through appropriate adjustment of the alarm threshold, enabling the system to maintain a relatively accurate and reliable overload alerting performance.

### 4.6. Effectiveness Evaluation of Self-Calibration Module

To quantitatively assess the impact of the car-base rubber aging on overload warning thresholds and verify the mitigation effect of self-calibration on premature false alarms, an experiment was designed with the following procedure: Load masses $F_{max}=800$ kg into the empty and stable car to calibrate the absolute height as the original overload threshold ($H_{th}$), then, unload the mass. Compress the car-base rubber under preloads $F_{pre}\in \left\{400,600,800\right\}$ kg for 17 continuous hours per load to induce material fatigue. After complete unloading, reload the car until reaching height $H=H_{th}$; then, record the actual load as $F_{alarm}$, and calculate the error $AE_{before}=\left|F_{max}-F_{alarm}\right|$. Activate the self-calibration protocol to generate a compensated threshold $H_{th}^{\prime }$. Reload the car to height $H=H_{th}^{\prime }$, record the calibrated load $F_{alarm}$, and calculate the residual error $AE_{after}=\left|F_{max}-F_{alarm}^{\prime }\right|$. The evaluation metrics are described as follows:

- Premature Alarm Rate (PAR)
  (16) $$PAR=\frac{F_{\max}-F_{\mathrm{alarm}}}{F_{\max}}\times 100\%.$$
  This quantifies severity of premature warnings (higher values indicate worse performance).
- Calibrated Residual Alarm Rate (CRAR)
  (17) $$CRAR=\frac{F_{\max}-F_{\mathrm{alarm}}^{\prime }}{F_{\max}}\times 100\%.$$
  This measures the residual error after calibration.
- Calibration Improvement Rate (CIR)
  (18) $$CIR=\frac{PAR-CRAR}{EAR}\times 100\%.$$
  This quantifies the mitigation efficacy (higher values indicate better calibration).

As evidenced by the data in Table 3, a higher preload $F_{pre}$ correlates directly with increased car-base rubber aging, resulting in a larger $AE_{before}$ (e.g., reaching 99 kg at $F_{pre}=800$ kg). This trend suggests that sustained high-load conditions significantly accelerate the elastic degradation of the rubber material, causing a substantial drift in the originally set $H_{th}$. This drift increases the likelihood of early triggering of the overload warning mechanism, potentially resulting in frequent false alarms during normal operation. Following self-calibration, these errors decreased significantly by 31 kg, 38 kg, and 59 kg, respectively, demonstrating more pronounced improvement under severe aging conditions.

As shown in Table 4, the EAR rises significantly with increasing aging severity, peaking at 12.38%. In contrast, the CRAR is successfully constrained below 5% across all cases. Furthermore, the CIR consistently exceeds 50%, reaching a maximum of 75.6%, highlighting the mechanism’s sensitivity and corrective capability even under mild fatigue conditions.

Overall, the experimental results confirm that the self-calibration function provides stable and substantial mitigation of premature overload warnings caused by car-base rubber fatigue. It effectively reduces the risk of overload misjudgments due to material aging, thereby validating the reliability, technical soundness, and engineering applicability of the proposed self-calibration module.

## 5. Conclusions and Outlook

This study presents an intelligent elevator weighing and warning system based on multimodal sensing, integrating draw-wire displacement sensing with visual perception. The core innovations encompass a load estimation algorithm, vision-based passenger recognition, and a self-calibration mechanism for mitigating overload false alarms. The comprehensive experimental validation demonstrates the following:

- 98.9% accuracy in identifying empty states;
- 93.5% precision for passenger counting;
- <5% maximum load estimation error relative to the rated capacity;
- ≤5% residual false alarm rate after self-calibration implementation.

This system achieves the real-time intelligent perception of cabin load status and passenger occupancy, effectively resolving the industry-wide challenge of premature overload warnings caused by car-base rubber aging.

Despite these advancements, certain limitations warrant further investigation:

- The visual recognition module exhibits reduced robustness under strong specular reflections and extreme occlusion scenarios, necessitating enhanced generalization capabilities.
- The load estimation accuracy experiences minor degradation during rapid passenger ingress/egress cycles, suggesting potential optimization in dynamic modeling.

These findings establish a critical technological pathway for multimodal data fusion and real-time inference on resource-constrained embedded platforms. The proposed methodology significantly elevates elevator operational safety and transport efficiency while providing a practical foundation for intelligent safety maintenance within the vertical transportation industry, thereby propelling the evolution of smart elevator systems.

## 6. Prospects

Future research will focus on improving the system’s long-term stability and adapting to complex operating environments. It will comprehensively promote material property analysis, visual perception optimization, and industry application verification to build a smart elevator monitoring and self-calibration system with higher precision, higher robustness, and scalable deployment.

- At the material level, we will work with the Materials Science Laboratory to conduct controlled experiments on the aging behavior of car-base rubber and collect deformation data on multiple elevators of the same model with different years of use over a long period of time to build an aging characteristic model and optimize the full life cycle compensation mechanism.
- In terms of visual perception, the detection categories will be expanded to cover large items and luggage, template matching will be introduced to optimize the recognition of “empty cabins”, and the YOLOv8 model will be trained with polarization filtering, adaptive thresholding, and reflection enhancement data to improve recognition capabilities in specular reflection and occlusion scenarios. At the same time, multi-view imaging and video sequence analysis will be explored to alleviate occlusion ambiguity, and the model’s generalization performance will be enhanced through the expansion of diversified scene data.
- At the industry application level, we will conduct long-term on-site research with elevator manufacturers and maintenance companies to quantify changes in the maintenance efficiency, service time, and operation and maintenance costs under different operating conditions, comprehensively verify the technical and economic benefits of the system in actual operation, and provide data support for its promotion and application.

## Figures and Tables

**Table 1:** Accuracy statistics of visual module recognition.

Detection Type	Total Samples (Frames)	Correct Identification (Frames)	Accuracy (%)
Empty State Identification	804	795	98.9
Passenger Counting	1500	1402	93.5

**Table 2:** Evaluation metrics of Scenario 1.

Group	Mean AE (kg)	Max AE (kg)
1	9.11	30.30
2	16.74	47.18
3	16.90	47.45
4	15.45	44.39
5	11.19	34.72
6	9.39	30.77
7	14.30	42.05
8	12.24	37.43

**Table 3:** Results of self-calibration under different preloads.

Aging Preloads	Before Self-Calibration		After Self-Calibration	
$F_{\mathrm{pre}}$ (kg)	$F_{\mathrm{alarm}}$ (kg)	${\mathrm{AE}}_{\mathrm{before}}$ (kg)	$F_{\mathrm{alarm}}^{\prime }$ (kg)	${\mathrm{AE}}_{\mathrm{after}}$ (kg)
400	759	41	790	10
600	733	67	771	29
800	701	99	761	40

**Table 4:** Results of valuation metrics.

$F_{\mathrm{pre}}$ (kg)	PAR (%)	CRAR (%)	CIR (%)
400	5.13	1.25	75.6
600	8.38	3.63	56.7
800	12.38	4.88	60.6

## Data Availability

The dataset generated and analyzed in this study has been made publicly available. The ElevatorCabinVision Dataset can be accessed at: https://pan.baidu.com/s/1EFyo0_G_t3BlvBpemf-9zA?pwd=evds. This dataset supports passenger detection and empty cabin recognition research in intelligent elevator systems.
